# Evaluation of MRI for initial staging of esophageal cancer: the STIRMCO study

**DOI:** 10.1007/s00330-025-11549-6

**Published:** 2025-05-16

**Authors:** Vincent Levy, Mario Jreige, Laura Haefliger, Celine Du Pasquier, Camille Noirot, Anna Dorothea Wagner, Styliani Mantziari, Markus Schäfer, Naik Vietti-Violi, Clarisse Dromain

**Affiliations:** 1 Department of Radiodiagnostic and Interventional Radiology, Lausanne University Hospital and University of Lausanne, Lausanne, Switzerland; 2 Department of Nuclear Medicine, Lausanne University Hospital and University of Lausanne, Lausanne, Switzerland; 3 Department of Oncology, Lausanne University Hospital and University of Lausanne, Lausanne, Switzerland; 4 Department of Visceral Surgery, Lausanne University Hospital and University of Lausanne, Lausanne, Switzerland

**Keywords:** Esophageal neoplasms, Magnetic resonance imaging, Neoplasm staging

## Abstract

**Objectives:**

To compare the diagnostic accuracy of MRI and PET/CT combined versus standard staging methods (CT, endoscopic ultrasound [EUS], and PET/CT) for initial staging of esophageal cancer (EC).

**Materials and methods:**

This study included patients newly diagnosed with histologically proven EC between 2017 and 2021. Patients underwent a 3-T esophageal MRI alongside standard staging (CT, EUS, PET/CT) prior to treatment. TNM-stages were assessed by two independent reviewers for MRI, CT, and PET/CT, with EUS evaluated by one operator. Discrepancies were resolved by a third reviewer. Patients were categorized based on treatment management: surgery (T1-T2N0M0), neoadjuvant (radio)chemotherapy (T3-T4a and/or N1-N2-N3M0), and palliative chemotherapy (T4b and/or M1). The reference standard was histopathology from surgical specimens or TNM staging from tumor board discussions. The area under the curve (AUC) was calculated for each imaging combination.

**Results:**

60 patients newly diagnosed with EC (50M/10F; mean age 66.5 years) were prospectively enrolled. MRI + PET/CT combination exhibited the highest AUC (0.92, 95% CI: 0.79–1) for differentiating curative versus palliative patients, without statistically significant difference compared to CT + EUS (0.80, 95% CI: 0.56–1, *p* = 0.34), CT + PET/CT (0.77, 95% CI: 0.53–1, *p* = 0.42), and CT + EUS + PET/CT (0.78, 95% CI: 0.58–0.97, *p* = 0.26). In term of differentiating patients eligible for upfront surgery from those with indication for neoadjuvant (radio)chemotherapy, the combination of CT + EUS + PET/CT demonstrated the highest AUC (0.90, 95% CI: 0.75–1) without statistically significant difference compared to CT + EUS (0.82, 95% CI: 0.56–1, *p* = 0.49), CT + PET/CT (0.79, 95% CI: 0.46–1, *p* = 0.36), and MRI + PET/CT (0.83, 95% CI: 0.65–1, *p* = 0.59).

**Conclusion:**

MRI + PET/CT combination is highly accurate for initial EC staging and non-inferior to standard methods, offering less invasiveness and reduced radiation exposure.

**Key Points:**

***Question*** *Can MRI help improve the TNM staging of esophageal cancer?*

***Findings*** *MRI + PET/CT showed no statistically significant difference compared to endoscopic ultrasound (EUS) + CT + PET/CT in identifying curative vs palliative patients but with a tendency for improved staging*.

***Clinical relevance*** *Thoraco-abdominal MRI can provide added value (as a replacement of CT and EUS) in initial staging of esophagus cancer, particularly in cases of stenotic or advanced tumors.*

**Graphical Abstract:**

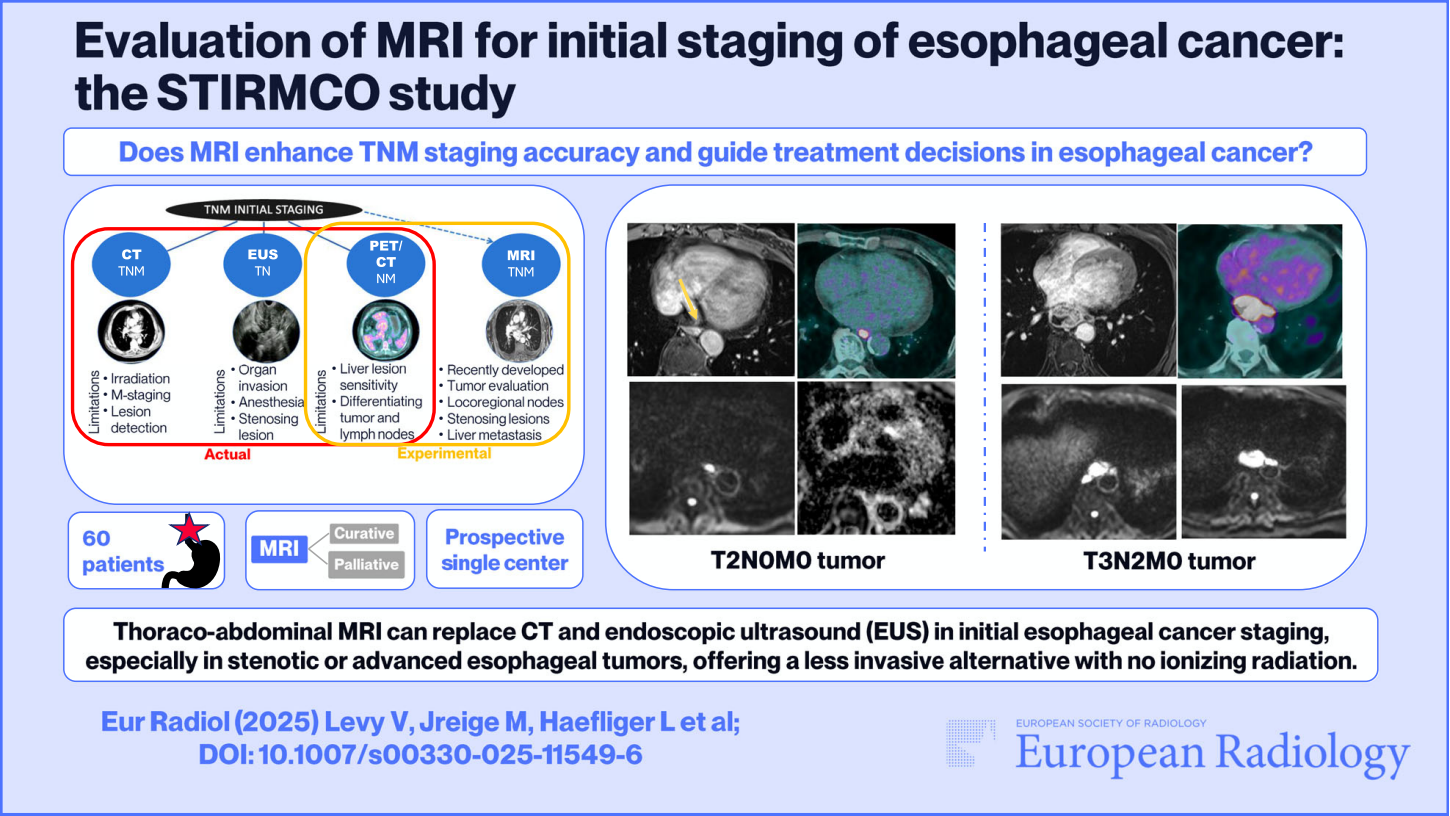

## Introduction

Esophageal cancer (EC), the 6th leading cause of cancer-related mortality and the 7th most prevalent cancer globally [1, 2], presents a poor prognosis with a 5-year survival rate of 12% [3], particularly affecting younger individuals under 50 at advanced stages [4], and is primarily divided into squamous cell carcinoma (linked to alcohol and tobacco) [5, 6] and adenocarcinoma (associated with gastro-esophageal reflux and obesity) [7, 8].

Accurate initial staging is crucial for determining the therapeutic strategy. For patients with early-stage EC, endoscopic resection, or surgery alone may be options [9]. Definitive chemoradiotherapy for squamous cell carcinoma, or neoadjuvant treatment (NAT), including preoperative chemoradiotherapy or perioperative chemotherapy, is indicated for locally advanced tumors. Metastatic tumors are treated with palliative intent using chemotherapy ± immunotherapy [10, 11]. Tumor extension assessment follows the 8th edition of the UICC-AJCC TNM staging system [12]. Currently, multiple imaging modalities are used, including a combination of contrast-enhanced CT, PET/CT and endoscopic ultrasound (EUS) [13–15]. CT provides detailed anatomical information and contributes to the T, N, and M staging, particularly in assessing the presence or absence of lung metastases. EUS evaluates local invasion and nearby structures (T staging) and local lymph nodes (N staging), whereas PET/CT offers valuable and crucial insights for the N and for M staging in surgical cases. Nevertheless, the current combination has inherent limitations, as both CT and PET/CT involve radiation, and about one-third of EC cases involve substantial stenosis, restricting EUS utilization [16–19].

Despite its numerous advantages, such as the absence of ionizing radiation, multiplanar imaging, high contrast resolution, functional imaging and improved soft tissue differentiation, the use of MRI for EC staging is not yet a routine method. However, recent studies have demonstrated that MRI is a potential alternative to EUS for locoregional tumor staging, showing high precision and good reproductivity [18, 20–24]. According to a recent meta-analysis, the sensitivity and the specificity of MRI for T-stage (T1/T2 vs T3/T4) are both 86% and 95%, while for N-stage, the sensitivity is 71% with a specificity of 72% [25]. A key advantage of MRI is that, like CT and PET/CT, it can be performed in cases of stenotic tumors, unlike EUS. Furthermore, MRI is less invasive than EUS and does not require general anesthesia [17]. Consequently, the American College of Radiology suggests that MRI “may be appropriate” for newly diagnosed EC [24].

The primary objective of our study was to compare the diagnostic performance of conventional procedures (EUS, CT, and PET/CT) with a new combination (MRI and PET/CT) for the initial staging of EC. Secondary objective was to assess the diagnostic performance of each procedure.

## Materials and methods

### Study design

This prospective single-center study, approved by the local institutional review board and ethics committee (STIRMCO protocol [STaging IRM Cancer Oesophageal], CER-VD 2017-00388), involved patients who provided informed signed consent before inclusion. The inclusion period was from October 2017 to December 2021. Eligible patients were adults (≥ 18 years) with newly diagnosed, histologically proven EC, including gastro-esophageal junction cancers. Exclusion criteria were cervical EC (primarily because their treatment options are non-surgical, leading to a different importance of TNM staging compared to other esophageal cancers), previously treated, pregnant women, and those with a contraindication for MRI. All included patients underwent a thoraco-abdominal MRI examination in addition to the standard staging procedures (EUS, CT, and PET/CT) before treatment initiation. Demographic, clinical, and pathologic data were retrieved from the patient’s medical records.

### Image acquisition

We conducted all MRI examinations using a 3-Tesla scanner (Magnetom PrismaFit 3 T, VE11E software version, Siemens Healthineers), except for one patient (1.5 T) because of machine availability (parameters are presented in Table 1). MRI sequences included sagittal and axial T2-weighted imaging Blade covering the thorax and whole liver, gated axial T2-weighted imaging turbo spin echo, axial diffusion-weighted imaging (DWI) (b50, 400, 800 s/mm^2^) with corresponding ADC map covering the thorax and whole liver and axial T1-weighted imaging Volume Interpolated Breath-hold Examination dixon before and after Gadolinium injection (Dotarem® 0.5 mmol Gd/mL, Guerbet). Dynamic acquisitions included arterial (20 s post-injection), portal venous (35 s post-injection), and delayed phases focused on the tumor (2 min post-injection), as well as an additional delayed phase of the upper abdomen covering the entire liver (7 to 8 min post-injection). The median acquisition time, approximately 31 min, varied based on patient breathing and tumor size. CT and FDG-PET/CT examinations were conducted as part of routine clinical practice. EUS was routinely conducted under general anesthesia by 6 gastroenterologists.

**Table 1:** MRI sequences parameters

	T2WI TSE Blade	T2WI TSE Blade	DWI chest	DWI liver	Dynamic T1WI VIBE Dixon
Plane	Axial	Sagittal	Axial	Axial	Axial
Volume	Chest and liver 2 boxes	Chest	Chest	Liver	Chest and liver 2 boxes
Repetition time TR (ms)	2290	2020	3500	2500	4.5
Echo time TE (ms)	68	89	59	59	1.35–2.58
Slice thickness (mm)	5	3	5	5	2.3
Field of view (mm)	371 × 371	400 × 400	380 × 261	380 × 261	350 × 273
Phase encoding	x (Radial)	x (Radial)	A > P	A > P	A > P
Matrix size	320	320	134 × 134	134 × 134	320 × 240
Voxel size (mm)	1.2 × 1.2 × 5	1.3 × 1.3 × 3	1.4 × 1.4 × 5 (interpolated)	1.4 × 1.4 × 5 (interpolated)	1.1 × 1.1 × 2.3
Number of slices	45	30	40	28	88
Distance factor (mm)	1	0.3	1	1	0.46
Flip angle (degree)	100	120	x	x	9
Acceleration factors	Grappa 3	Grappa 2	SMS 4	SMS 4	Caipirinha 4
Turbo factors (or EPI factor)	35	31	92	92	x
Bandwidth (Hz/Px)	781	1563	2332	2332	1040
Nex	1	1	1	1	1
Acquisition time (min)	1 min 23 s	1 min 51 s	1 min 43 s	1 min 14 s	18 s/phase
Number of apneas	4	4	FB	FB	4

Two 16-channel body array coil and a 32-channel spine coil (Siemens Healthcare) were employed. Patients were positioned supine with hearing protection

*TSE* turbo spin echo, *DWI* diffusion-weighted imaging, *VIBE* volume interpolated breath-hold examination, *TR* repetition time, *TE* time to echo, *EPI* echo planar imaging, *Hz/Px* Hertz/pixel, *FB* free breathing

### Image analysis

A reading sheet was created for the study for each modality to determine the initial TNM stage for each patient according to the 8th edition UICC-AJCC TNM classification [12]. The remaining parameters on the form included the tumor’s location and t size as assessed by EUS, CT and MRI.

Readers were asked to analyze the T stage on CT, EUS, and MR images, the N stage on CT, EUS, MR and PET/CT images, and the M stage on CT, PET/CT, and MR images. Except for EUS, images from each modality were independently reviewed by two different readers specialized in oncologic and gastrointestinal imaging (L.H. and V.L. for CT with 5 and 3 years of experience, respectively; C.D. and C.D.P. for MRI with 25 and 6 years of experience respectively and L.H. and C.N., both with 5 years of experience). Axial and sagittal T2-weighted images were used to determine tumor location. Chest DWI images were used for mediastinal lymph nodes staging (N staging). DWI and delayed phase images on the liver were used to detect liver metastasis (M staging) and abdominal lymph nodes (N staging). Finally, axial T2W and dynamic T1W images were collectively used for T staging and mediastinal N staging. The EUS form was completed by the gastroendoscopist who conducted the EUS. The initial reading was conducted prospectively at the time of each examination, and a second reading was performed retrospectively 3 months after the inclusion of the last patients, independently of the initial readings. In cases of discrepancies between the two readings, a consensus was reached by a third reader. All data are provided in the Supplementary Material.

### Reference standard

The patients were classified into two categories: those who underwent upfront surgery, for whom the gold standard was the histopathological analysis of the resection specimen, and those who received treatment before surgery or were treated non-surgically, for whom the gold standard was the TNM classification obtained during the TBM (TNM_TBM_). This TNM_TBM_ included data from all imaging, endoscopic procedures and pathological analysis performed prior to treatment. It encompassed biopsies of suspected lymph nodes and bronchoscopy with endobronchial ultrasonography in cases of suspected airway involvement. This TNM_TBM_ classification served as the reference for the therapeutic management of the patient.

### Statistical analysis

Demographic and clinical characteristics were expressed as frequency (%) for categorical variables and median with 95% confidence interval (CI) or mean and standard deviation (SD) for continuous variables. We evaluated interreader agreement using Cohen’s Kappa coefficient (K) for CT, PET/CT and MRI.

We evaluated the individual diagnostic performance of CT, PET/CT, EUS and MRI for the initial TNM staging of EC compared to the reference standard. This involved calculating sensitivity, specificity, positive predictive value, negative predictive value, and the AUC for each technique in distinguishing between non-advanced tumors (T1-T2-T3) versus advanced tumors (T4) compared to the reference standard. This process was repeated to compare cancer with lymph node involvement (N0) versus those with lymph node involvement (N1-2-3) and non-metastatic tumors (M0) versus metastatic tumors (M+). To compare the diagnostic performance of the imaging modalities, we used a test for equality of two or more ROC areas to assess differences in AUCs using the DeLong test for ROC curves.

We also examined different staging strategies corresponding to 4 combinations of different modalities: (1) CT + EUS + PET/CT; (2) CT + EUS; (3) CT + PET/CT; (4) MRI + PET/CT. For that, patients were subsequently categorized into 3 groups based on the treatment strategy. (1) patients scheduled for surgery (early stage: T1-T2N0M0), (2) patients eligible for NAT or definitive (radio)chemotherapy (intermediate stage: T3-T4a and/or N1-N2M0), and (3) patients with metastatic or unresectable tumors, designated for palliative treatment (T4b and/or M1). Our assessment focused on differentiating between patients scheduled for upfront surgery and those receiving other treatments (group 1 versus groups 2 + 3) and evaluating the discrimination among curative and palliative patients (groups 1 + 2 versus group 3). All statistical analyses were performed using STATA version 18.0 (STATA Corp.). *p*-values < 0.05 were considered as statistically significant.

## Results

### Patient population

**Fig. 1:**
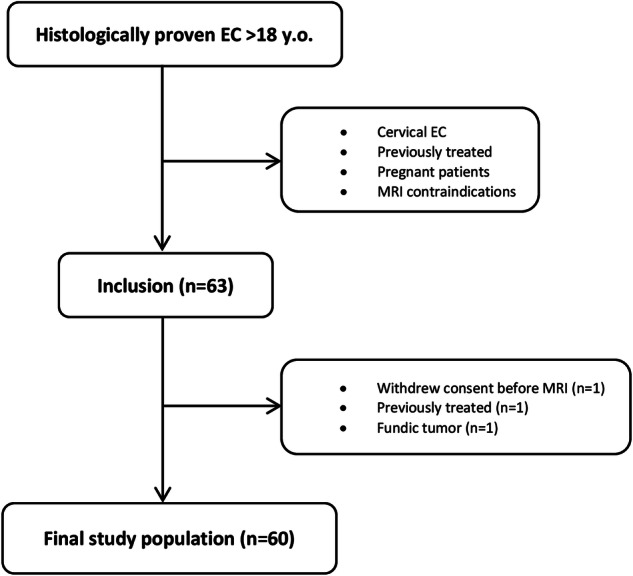
Flowchart. EC, esophageal cancer

Sixty-three patients were prospectively included in the STIRMCO study. Three patients were excluded from this study. Consequently, we analyzed a total of 60 patients (Fig. 1). Our study population consisted of 10 females and 50 males, with a mean age of 66.5 years (range: 41–83 years). The predominant histological type was adenocarcinoma (55%). A significant number of patients present dysphagia upon diagnosis, including 9 patients (15%) with dysphagia for fluids and 39 patients (65%) with dysphagia for solids. Among the patients of this study, 7 underwent upfront surgery without NAT, 34 received NAT followed by surgery, 12 received definitive radiochemotherapy, and 7 received palliative chemotherapy. Patient data, tumor characteristics, and procedure details are presented in Table 2.

**Table 2:** Patients and tumor characteristics

Patients (*n* = 60)	*n* (%)
Mean age in years (range)	66.5 (41–83)
Sex *n* female (%)/*n* male (%)	10 (16.7)/50 (83.3)
Body mass index in kg/m^2^ (range)	25.6 ± 9.4 (13.8–38.9)
Chronic alcohol consumption, *n* (%)	
Active	27 (45)
Past/absent	33 (55)
Smoking status, *n* (%)	
Active	23 (38.3)
Past/absent	37 (61.7)
Symptoms, *n* (%)	
Absent	12 (20)
Dysphagia for fluid	9 (15)
Dysphagia for solids	39 (65)
Treatment, *n* (%)	
Upfront surgery	7 (11.7)
NAT followed by surgery	34 (56.6)
Definitive radiochemotherapy	12 (20)
Palliative chemotherapy	7 (11.7)
Analyzable examinations, *n* (%)	
PET/CT	55 (91.7)
CT	45 (75)
EUS	56 (93.3)
MRI	60 (100)
Histological type, *n* (%)	
Adenocarcinoma	33 (55)
Squamous cells	25 (41.7)
Neuroendocrine	2 (3.3)
Histologic grade, *n* (%)	
Grade 1	4 (6.7)
Grade 2	30 (50)
Grade 3	26 (43.3)

*n* number, *PET/CT* positron emission tomography with [18F]2-fluoro-2-deoxy-D-glucose computed tomography, *EUS* endoscopic ultrasound

Among the 60 patients in the study, data were available for all (100%) for MRI, 56 for EUS (93.3%), 55 for PET/CT (91.7%) and 45 for CT (75%). CT and PET/CT examinations were performed as routine clinical exams across various centers in Switzerland, with differing protocols and equipment. 45 CT examinations ranged from 32 to 320 slices and were all performed with the injection of iodinated contrast media. Protocols varied among centers based on the specific objectives addressed in the CT examination. For 7 among the 45 patients, contrast-enhanced CT images were acquired during the PET/CT scan, corresponding to an additional acquisition with iodinated contrast agent injection ensuring diagnostic quality. The 55 PET/CT examinations were performed approximately 60 min after intravenous injection of a planned 2 to 3.5 MBq/kg of 18F-FDG. All patients fasted for at least 6 h before the scan. A low-dose helical CT scan was performed in all patients for anatomical correlation and attenuation correction. Of the 4 patients without EUS data, 2 did not undergo the exam and 2 were unable to due to stenotic tumor. One of the 56 patients had two synchronous tumors, with the stenotic tumor preventing EUS exploration of the second. For PET/CT, 4 patients did not undergo the exam, and 1 did not complete it as metastasis involvement had already been diagnosed on CT.

### Interreader agreement

Interreader agreement was strong to almost perfect for all imaging techniques, including MRI (K range: 0.70–0.90). MRI exhibited the highest interreader agreement for both T staging (K = 0.88, 95% CI: 0.77–0.99) and M staging (K = 0.82, 95% CI: 0.56–1) without statistically significant difference when considering the 95% confidence intervals. CT demonstrated higher interreader agreement than MRI for N staging, without statistically significant difference in Table 3.

**Table 3:** Interreader agreement

Initial staging	PET/CT K (95% CI)	CT K (95% CI)	MRI K (95% CI)
T		0.79 (0.62–0.96)	0.88 (0.77–0.99)
N	0.70 (0.53–0.87)	0.90 (0.80–1)	0.72 (0.58–0.85)
M	0.78 (0.48–1)		0.82 (0.56–1)

*T* tumor, *N* node, *M* metastasis, *K* Cohen’s Kappa coefficient, *PET/CT* positron emission tomography with [18F]2-fluoro-2-deoxy-D-glucose computed tomography

The empty cells represent “not applicable” (n/a) for PET/CT, as T staging is not assessed with this modality. The agreement level was interpreted as poor (K < 0), slight (0 ≤ K ≤ 0.2), fair (0.2 < K ≤ 0.4), moderate (0.4 < K ≤ 0.6), almost perfect (0.6 < K ≤ 0.8), or perfect (K > 0.8)

### Diagnostic performance of each imaging modality

**Fig. 2:**
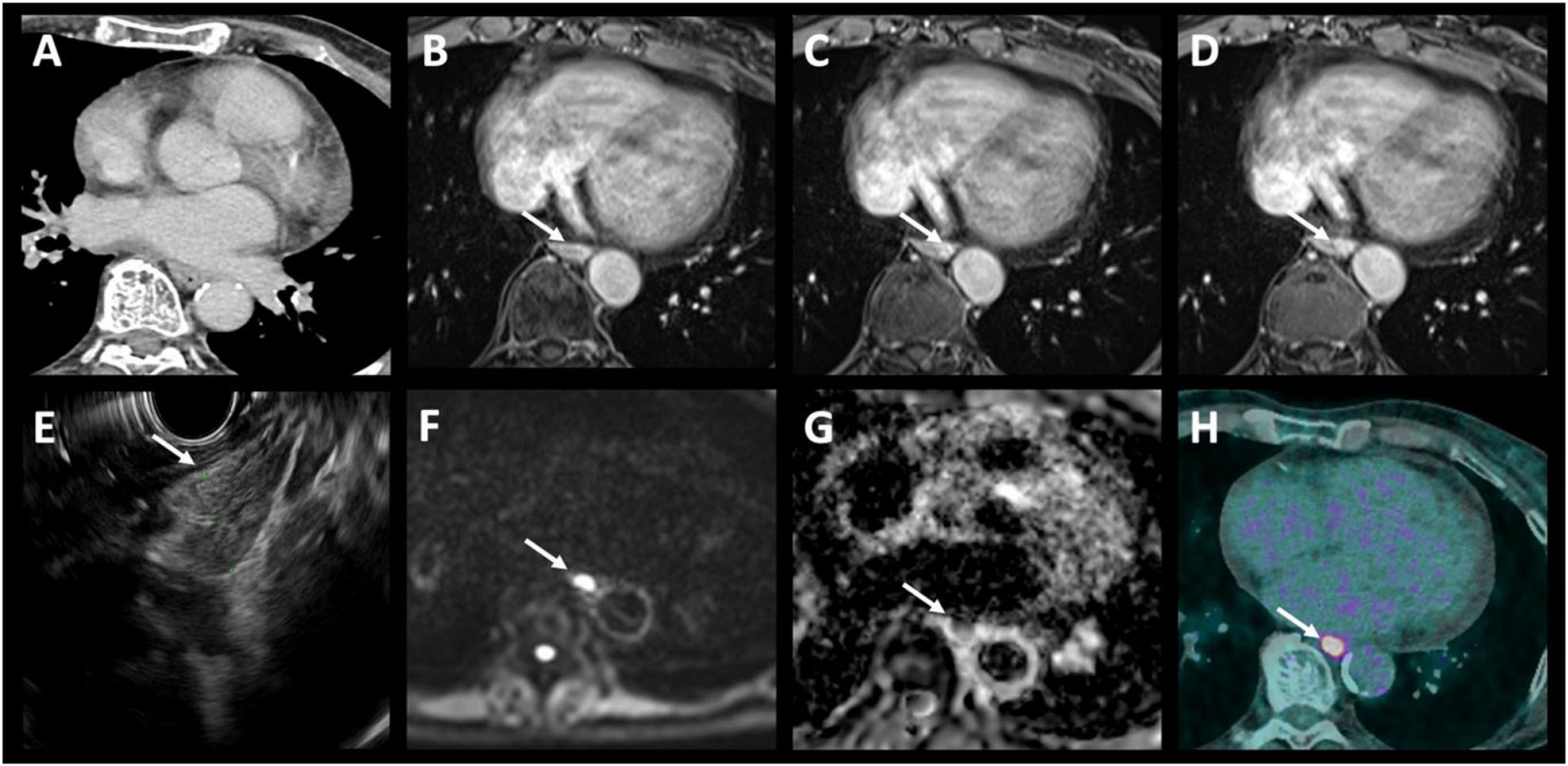
Example 2—Small tumor not detectable on CT. A 69-year-old patient with squamous cell carcinoma located in the middle third of the esophagus. The contrast-enhanced CT image (**A**) does not show the tumor. Contiguous enhanced-T1 MR images (**B**–**D**) show a small lesion on the posterolateral wall of the esophagus with restricted diffusion (**F**, **G**), classified T2N0. The T2 stage was confirmed by endoscopic ultrasound (**E**), and the absence of adenopathy and distant metastases was confirmed by PET/CT images (**H**)

**Fig. 3:**
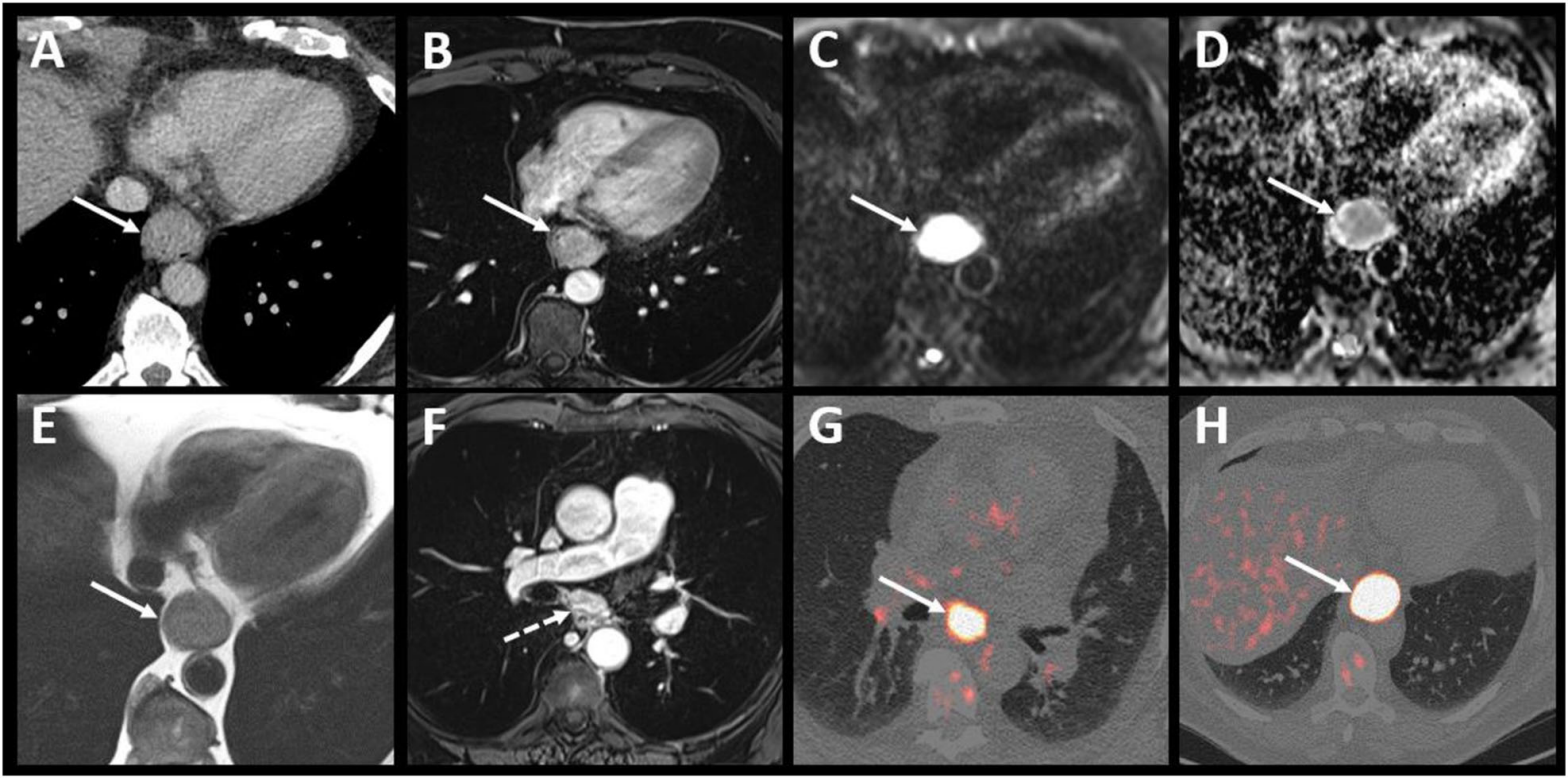
Example 3—High-quality soft tissue contrast in MR. A 60-year-old patient with adenocarcinoma located in the middle third of the esophagus, with CT image (**A**) suggesting a possible pleural invasion (white arrow). T1-enhanced MR (**B**) and diffusion-weighted (**C**) with ADC map (**D**) images clearly depict a circumferential esophageal tumor. The T2 MR image (**E**) shows a thin, fat interface between the tumor and the pleura, allowing pleural invasion to be ruled out, thus classifying the tumor as T3. T1-enhanced MR image (**F**) also depicted a small adenopathy in contact to the tumor (dotted arrow) classified the tumor as N1. This small lymph node was difficult to visualize on PET/CT images (**G**, **H**) due to the overlap of tracer fixation between the primary tumor and the adenopathy

The sensitivity, specificity, and diagnostic accuracy evaluated using the area under the curve (AUC) of each modality for TNM staging are presented in Table 4. MRI had the highest sensitivity and accuracy to identify T1-T3 versus T4 tumors, with a sensitivity of 79% (95% CI: 0.49–0.95), a specificity of 87% (0.74–0.95) and an AUC of 0.83 (0.71–0.95) without statistically significant difference compared to CT (0.74, 95% CI: 0.58–0.9, *p* = 0.23) and EUS (0.80, 95% CI: 0.66–0.93, *p* = 0.79) (Figs. 2, 3).

**Table 4:** Diagnostic performance of each modality

Initial staging	Sensitivity % (95% CI)				Specificity % (95% CI)				Area under the curve *n* (95% CI)			
	PET	CT	EUS	MRI	PET	CT	EUS	MRI	PET	CT	EUS	MRI
T1-T3 vs T4		54.5 (0.23–0.83)	64.3 (0.35–0.87)	78.6 (0.49–0.95)		94.1 (0.80–0.99)	95.2 (0.83–0.99)	87 (0.74–0.95)		0.74 (0.58–0.9)	0.80 (0.66–0.93)	0.83 (0.71–0.95)
N0 vs N+	67.4 (0.51–0.81)	94.4 (0.81–0.99)	89.1 (0.76–0.96)	87.5 (0.75–0.95)	100 (0.75–1)	55.6 (0.21–0.86)	66.7 (0.30–0.93)	69.2 (0.34–0.91)	0.84 (0.77–0.91)	0.75 (0.58–0.93)	0.78 (0.61–0.95)	0.78 (0.65–0.92)
M0 vs M+	100 (0.48–1)	40 (0.05–0.85)		100 (0.54–1)	100 (0.93–1)	100 (0.91–1)		100 (0.93–1)	1 (1–1)	0.7 (0.46–94)		1 (1–1)

The empty cells represent “not applicable” (n/a) as T staging is not assessed with PET/CT and M stage with EUS

*TNM* tumor, nodes, metastasis, *CI* confidence interval, *PET* positron emission tomography with [18F]2-fluoro-2-deoxy-D-glucose computed tomography, *CT* computed tomography, *MRI* magnetic resonance imaging

**Fig. 4:**
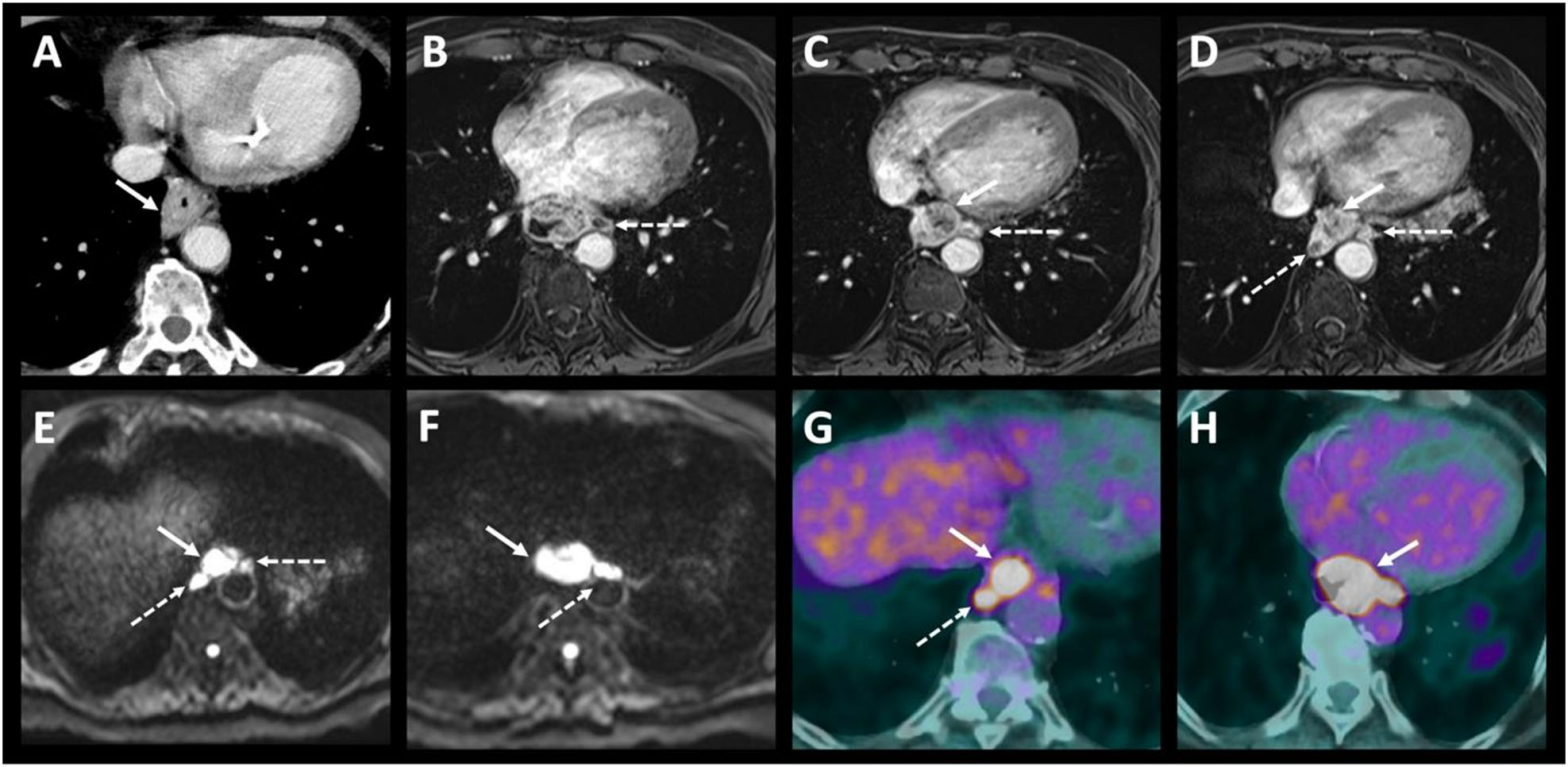
Example 3—MRI and PET/CT for lymph node evaluation. A 70-year-old patient with squamous cell carcinoma located in the lower third of the esophagus. Contrast-enhanced CT image (**A**) depicted the tumor without lymphadenopathy, while enhanced-T1 MR (**B**–**D**) and diffusion-weighted MR (**E**, **F**) images show the esophageal tumor (white arrows) but also multiple adjacent lymphadenopathies (white dotted arrows). The primary esophageal tumor (white arrow) and adjacent lymphadenopathies (white dotted arrows) were also clearly depicted on PET/CT images (**G**, **H**)

In differentiating tumors (N0) from those (N1-2-3), PET/CT had highest specificity of 100% (95% CI: 0.75–1) with low sensitivity of 67% (0.51–0.81) and the highest AUC of 0.84 (0.77–0.91) without statistically significant difference compared to CT (0.75, 95% CI: 0.58–0.93, *p* = 0.54), EUS (0.78, 95% CI: 0.61–0.95, *p* = 0.81) and MRI (0.78, 95% CI: 0.65–0.92, *p* = 0.61) (Figs. 3, 4).

**Fig. 5:**
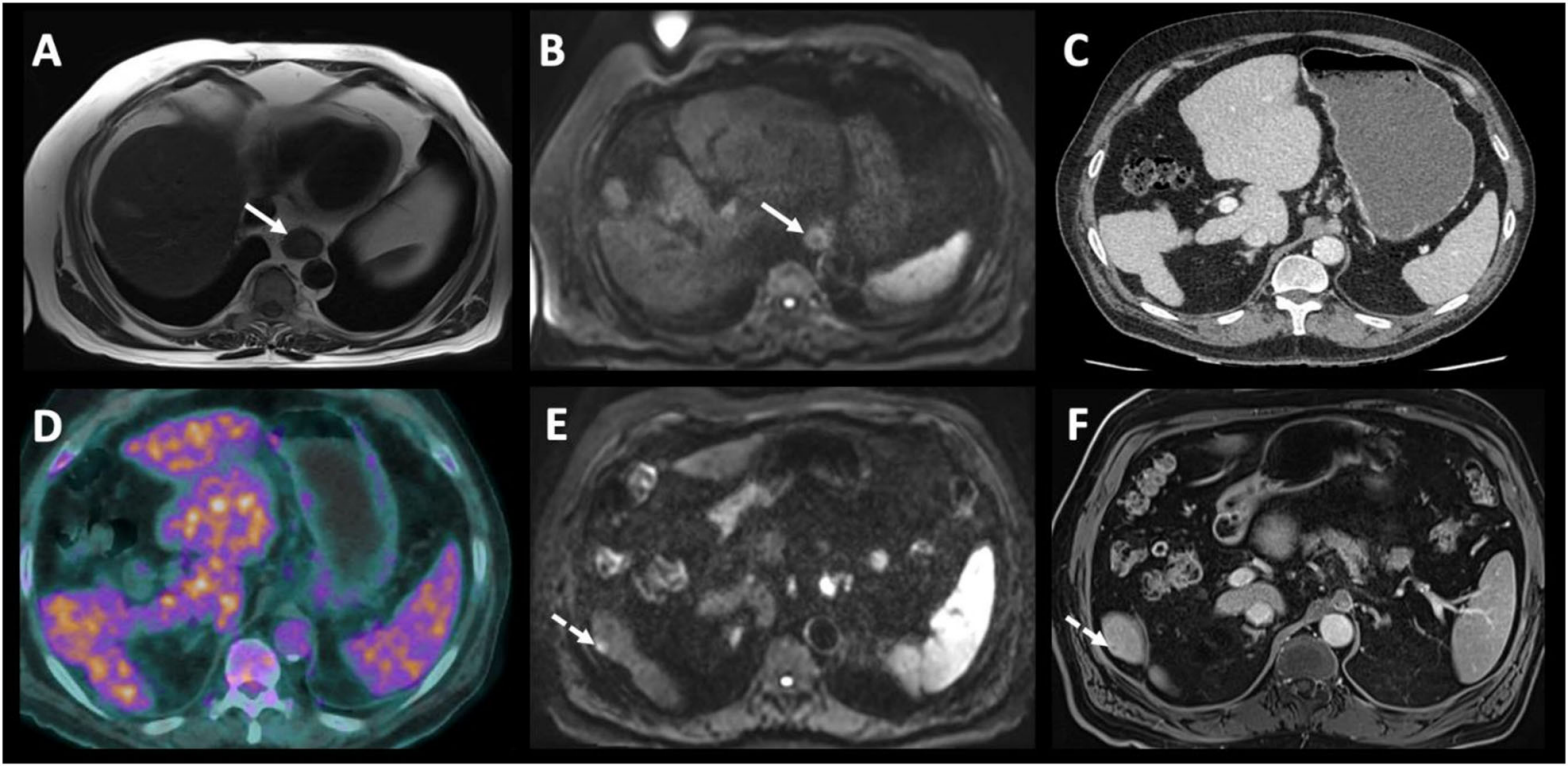
Example 4—Strengths of MRI for assessing liver metastases. Esophageal MRI evidenced the esophago-gastric tumor with intermediary signal on T2 (**A**, arrow) with restricted diffusion (**B**, arrow). The tumor was classified T3N2 based on MRI and echo-endoscopy (not presented). No metastasis was seen on CT and PET/CT (**C**, **D**). However, MRI evidenced a 7-mm liver lesion on segment VI with restricted diffusion (**E**, dotted arrow) and T1 hypo-intensity on portal venous phase (**F**, dotted arrow). Biopsy confirmed as a metastasis from esophago-gastric cancer

For discriminating between non-metastatic tumors (M0) and metastatic tumors (M+), the lowest sensitivity was for CT with a sensitivity of 40% (95% CI: 0.05–0.85). The AUC was perfect for MRI and PET/CT compared to CT (1, 95% CI: 1–1 versus 0.7, 95% CI: 0.46–0.94) (Fig. 5).

### Diagnostic performance of the different staging strategies

The sensitivity, specificity, and diagnostic accuracy evaluated using the area under the curve (AUC) of 4 combinations of staging procedures for TNM staging are presented in Table 5.

**Table 5:** Diagnostic performance of the four combinations of staging procedures

Combinations	Diagnostic performance Groups 1 + 2 vs group 3			Diagnostic performance Group 1 vs groups 2 + 3		
	Sensitivity % (95% CI)	Specificity % (95% CI)	AUC *n* (95% CI)	Sensitivity % (95% CI)	Specificity % (95% CI)	AUC *n* (95% CI)
CT + EUS	60 (0.15–0.95)	100 (0.99–1)	0.80 (0.56–1)	97.2 (0.86–0.99)	66.7 (0.09–0.99)	0.82 (0.56–1)
CT + PET/CT	60 (0.15–0.95)	94.4 (0.81–0.99)	0.77 (0.53–1)	92.1 (0.79–0.98)	66.7 (0.09–0.99)	0.79 (0.46–1)
CT + EUS + PET/CT	57.1 (0.18–0.90)	97.8 (0.88–0.99)	0.78 (0.58–0.97)	93.3 (0.82–0.99)	85.7 (0.42–0.99)	0.90 (0.75–1)
MRI + PET/CT	87.5 (0.47–0.99)	96.2 (0.87–0.99)	0.92 (0.79–1)	94.3 (0.84–0.99)	71.4 (0.29–0.96)	0.83 (0.65–1)

Group 1: T1-T2 N0 M0 (treatment: Upfront surgery), Group 2: T3-T4a and/or N1-N2-N3 M0 (treatment: Neoadjuvant (radio)chemotherapy) and Group 3: T4b and/or M1 (treatment: Palliative chemotherapy)

*TNM* tumor nodes metastasis, *CI* confidence interval, *PET/CT* positron emission tomography with [18 F]2-fluoro-2-deoxy-D-glucose computed tomography, *CT* computed tomography, *MRI* magnetic resonance imaging

To differentiate patients scheduled for surgery from those receiving other treatments (group 1 versus 2 + 3), the combination of EUS + CT + PET/CT demonstrated the highest AUC (0.90, 95% CI: 0.75–1) without statistically significant difference compared to CT + EUS (0.82, 95% CI: 0.56–1, *p* = 0.49), CT + PET/CT (0.79, 95% CI: 0.46–1, *p* = 0.36) and MRI + PET/CT (0.83, 95% CI: 0.65–1, *p* = 0.59).

To differentiate patients categorized as curative from those categorized as palliative (groups 1 + 2 versus 3), the AUC was the best for combination of MRI + PET/CT (0.92, 95% CI: 0.79–1, *p* = 0.36) without statistically significant difference compared to CT + EUS (0.80, 95% CI: 0.56–1, *p* = 0.36), CT + PET/CT (0.77, 95% CI: 0.53–1, *p* = 0.42) and CT + EUS + PET/CT (0.78, 95% CI: 0.58–0.97, *p* = 0.26).

## Discussion

Our study found that the combination of MRI + PET/CT is highly accurate for the initial staging of EC, non-inferior to current staging procedures CT + EUS + PET/CT, while being less invasive and involving less radiation exposure. MRI demonstrates high diagnostic accuracy statistically comparable to EUS and CT in differentiating T1-T3 and T4 esophageal tumors, as well as between N1 and N0 tumors. Furthermore, MRI exhibits significantly higher accuracy than CT in M staging. We also observed robust interreader agreement, ranging from strong to almost perfect (0.7–0.9), across all imaging techniques, including MRI, for TNM staging. Despite commonly being perceived as a complex technique requiring specialized expertise for interpretation, our study indicates that MRI interpretation shows a high level of reproducibility like that of CT and PET/CT. Notably, the substantial agreement between a senior and a junior radiologist in MRI interpretation underscores its reliability.

EC shows a gender disparity, with men at 2–8 times higher risk, primarily affecting older adults. 60% of cases are in those over 65, reflected in our cohort of 60 patients, consisting of 83.3% males and a predominance of adenocarcinoma (55%) [1].

MRI’s high soft tissue contrast, routinely used in rectal cancer staging, aids in distinguishing EC infiltrating periesophageal fat and nearby structures like the aorta, tracheobronchial tract, and diaphragm (Figs. 2, 3). This study’s results align with existing literature on MRI’s effectiveness [25]. Wang et al [26] explored the value of 3-T MRI for evaluating T staging of esophageal tumors after NAT and before surgery with pathological correlation. They found excellent interreader agreement in the MR-T staging. The greatest precision for T0, T1, T2, and T4a lesions was achieved with delayed phase StarVIBE (96.2–94.9%, 92.4–89.9%, 91.1–91.1%, and 91.1–94.9% for both readers, respectively), where T3 lesions were best identified using T2-weighted TSE BLADE (92.4% and 94.9%, respectively). Guo et al evaluated the diagnostic precision of 3-Tesla MRI in comparison to CT and EUS for preoperative T-staging of potentially operable EC [20]. The agreement between readers for pre- and post-operative T-staging was excellent across all imaging methods. MRI demonstrated significantly greater accuracy compared to CT for both readers. Additionally, MRI exhibited higher specificity and accuracy compared to EUS. Lee et al conducted a systematic review and meta-analysis to assess the diagnostic accuracy of MRI in staging esophageal carcinoma in patients prior to esophagectomy and pathological staging between 2000 and 2019. Twenty studies, including a total of 984 patients, were included. The pooled accuracy for distinguishing stage T0 from T1 or higher showed a sensitivity of 92% and a specificity of 67%. For distinguishing stage T2 or lower from T3 or higher, the pooled accuracy had a sensitivity of 86% and a specificity of 86%. The pooled accuracy for distinguishing stage N0 from stage N1 or higher had a sensitivity of 71% and a specificity of 72% [25].

However, MRI has limitations, such as claustrophobia in some patients and longer scan times than CT [27]. It is also prone to motion artifacts, and metal clips from esophageal biopsies can hinder examination quality. Despite this, MRI showed comparable diagnostic performance for lymph node involvement (N0 vs N+) compared to CT, EUS, and PET/CT. In our study, MRI also demonstrated similar diagnostic performance for lymph node involvement staging (N0 versus N+) compared to CT, EUS and PET/CT. It is worth noting the low sensitivity of PET/CT for lymph node involvement staging (0.67, 95% CI 0.51–0.81), likely due to the overlap of lymph node and primary tumor hypermetabolism, which limits the detection of small local lymph nodes (Fig. 3). MRI’s high soft tissue resolution and multi-sequence enable precise differentiation between tumors and lymph nodes (Fig. 4). This was previously demonstrated by Shuto et al, who found that DWI is more sensitive than PET/CT in detecting lymph nodes from squamous cell carcinoma [28]. These results raise questions about EUS, currently seen as the most accurate method for TNM staging of EC [18]. However, this is an invasive procedure, operator dependent with limited value in case of tumor stricture [17, 29], as well as when multiple tumors are present, as demonstrated in our study where the evaluation of a second lesion was hindered by a stenotic lesion. Schreurs et al explored the individual contributions of EUS, CT, and PET/CT in the optimal staging sequence for EC in 216 operable patients. They found a nonsignificant impact of EUS after PET/CT or CT in classifying tumors as either resectable with curative intent or incurable/unresectable [19].

The lowest sensitivity observed in our study in discriminating between non-metastatic and metastatic tumors was noted for CT, aligning with existing literature. Rice et al reported that CT can detect metastasis with a sensitive range of 37–66% [30]. In our study, CT missed hepatic metastases in three patients that MRI detected. One lesion was also overlooked on PET/CT due to its small size and normal liver uptake. This emphasizes the need for dedicated abdominal scans during MRI assessments for EC. Our protocol includes T2-weighted imaging (T2WI), DWI, and post-contrast T1-weighted imaging (T1WI) to improve liver metastasis detection, which is common in EC.

In our study, we tested various imaging combinations, including MRI, to determine the optimal treatment strategy. For distinguishing patients eligible for curative treatment (T1-T4a and M0) from those needing palliative care (T4b and/or M+), the MRI + PET/CT combination showed the highest accuracy, though not significantly better than other combinations. To differentiate patients eligible for upfront surgery (T1-2 N0 M0) from those needing neoadjuvant (radio)chemotherapy (T3-T4 and/or N+ and/or M+), CT + EUS + PET/CT combination showed the highest accuracy, though not significantly better than MRI + PET/CT. MRI + PET/CT offers several advantages: (1) less reader dependency than EUS, (2) greater accuracy in stenosis cases, (3) reduced invasiveness compared to EUS, (4) no radiation exposure like CT, and (5) improved assessment of liver metastases.

The MRI + PET/CT combination, as effective as standard staging methods, offers a promising alternative to CT and EUS for initial EC staging. These complementary techniques enable detailed EC phenotyping: MRI assesses tumor cellularity (ADC) and soft tissue, while PET/CT evaluates metabolism (SUV) and detects distant metastases [31]. EUS and MRI combined are also useful in T-staging, with EUS for small tumors and MRI for stenotic or advanced tumors. This integrated approach is particularly effective for surgical planning, using MRI for local staging and liver metastasis (Fig. 5) and PET/CT for distant metastasis detection.

Our study shows promising results but is limited by a small, single-center cohort, incomplete data for CT, PET/CT and EUS conducted by 6 endoscopists, and the gold standard assessment being influenced by most patients NAT.

Although no lung metastases were found in our study, EC can spread to the lungs [32]. While CT is the gold standard for lung assessment, PET/CT in free breathing could limit parenchymal analysis [33]. This could be improved with a breath-hold sequence, and new MRI techniques like Utra-Short Echo Time (UTE) are being developed to enhance pulmonary nodule detection [30, 34]. These limitations highlight the need for larger multicenter studies to evaluate MRI + PET/CT as a substitute for standard procedures and to assess its cost-effectiveness in different countries.

## Conclusion

MRI provides high accuracy in the initial TNM staging of EC, facilitating the optimal evaluation of adjacent organ invasion. The combination of MRI + PET/CT for initial staging of EC is highly accurate and non-inferior to current staging procedures combination, with the advantage of being less invasive and involving less radiation exposure.

## Supplementary information

ELECTRONIC SUPPLEMENTARY MATERIAL

## Abbreviations

ADC: Apparent diffusion coefficient
AJCC: American Joint Committee on Cancer
AUC: Area under the curve
CT: Computed tomography
DWI: Diffusion weighted imaging
EC: Esophageal cancer
EUS: Endoscopic ultrasound
MRI: Magnetic resonance imaging
NAT: Neoadjuvant treatment
PET/CT: Positron emission tomography with [18F]2-fluoro-2-deoxy-D-glucose computed tomography
STIRMCO: STaging IRM Cancer Oesophageal
TNM: Tumor, node, metastasis
TSE: Turbo spin echo
UICC: Union for International Cancer Control
