# Supplementary material for: Evaluation of MRI for initial staging of esophageal cancer: the STIRMCO study
Source: Eur Radiol. 2025 May 16;35(11):6917–27. doi: 10.1007/s00330-025-11549-6 (PMC12559062; doi:10.1007/s00330-025-11549-6)
Supplement: Supplementary file 1 — ELECTRONIC SUPPLEMENTARY MATERIAL [file 330_2025_11549_MOESM1_ESM.pdf]

Evaluation of MRI for initial staging of esophageal cancer: The STIRMCO study.

ELECTRONIC SUPPLEMENTARY MATERIAL

Descriptive results of TNM staging of the 60 patients

The tables show the T, N and M staging assessed by two readers for MRI, CT and PET/CT, with consensus provided by a third reader in cases of discrepancies. Results for EUS are provided by the endoscopist who performed the examination. Among the 60 patients in the study, data were available for 100% of patients for MRI, 56 patients (93.3%) for EUS, 55 patients (91.7%) for PET/CT and 45 patients (75%) for CT. N+ = Used in EUS when the report indicates the presence of adenopathy without specifying the number, n/a = Not applicable.

T STAGE							
Patients	MRI			CT			EUS
	Reader 1	Reader 2	Consensus	Reader 3	Reader 4	Consensus	
1	T4b	T4b	-	T4b	T4b	-	T4a
2	T4a	T4a	-	T3	T3	-	T3
3	T4a	T4a	-	T4a	T4a	-	T3
4	T4a	T4a	-	T4a	T4a	-	T4b
5	T3	T3	-	T3	T3	-	T3
6	T4a	T4a	-	n/a	n/a	n/a	T3
7	T3	T3	-	T3	T3	-	T2
8	T4b	T4b	-	n/a	n/a	n/a	T4b
9	T2	T3	T2	T2	T2	-	T2
10	T4a	T4a	-	T4a	T4a	-	T3
11	T3	T3	-	T3	T3	-	T3
12	T2	T2	-	n/a	n/a	n/a	T2
13	T2	T2	-	n/a	n/a	n/a	T2
14	T2	T2	-	T2	T2	-	T1b
15	T3	T3	-	T2	T3	T3	T3
16	T3	T3	-	T2	T2	-	T2
17	T4a	T4a	-	T3	T3	-	T3
18	T4a	T4a	-	T3	T3	-	T3

19	T2	T2	-	n/a	n/a	n/a	T2
20	T2	T2	-	n/a	n/a	n/a	T4a
21	T3	T4a	T3	T3	T3	-	T3
22	T4b	T4b	-	T3	T3	-	T4a
23	T4a	T4a	-	T4a	T4a	-	T3
24	T3	T4a	T3	n/a	n/a	n/a	T3
25	T3	T3	-	T3	T3	-	T3
26	T3	T3	-	n/a	n/a	n/a	T3
27	T3	T3	-	T3	T3	-	T3
28	T3	T3	-	n/a	n/a	n/a	T3
29	T3	T3	-	T3	T3	-	T3
30	T4b	T4b	-	T2	T3	T3	T3
31	T3	T3	-	T2	T2	-	T2
32	T3	T3	-	T4a	T3	T3	T3
33	T3	T3	-	T3	T2	T3	n/a
34	T3	T3	-	T3	T3	-	T3
35	T3	T3	-	T3	T3	-	T3
36	T4a	T4a	-	T4a	T4a	-	T4a
37	T3	T3	-	T3	T3	-	T2
38	T3	T3	-	T3	T4a	T4a	T3
39	T2	T2	-	n/a	n/a	n/a	T3
40	T3	T3	-	n/a	n/a	n/a	T3
41	T4b	T4b	-	T3	T4a	T4a	T4a
42	T2	T2	-	T2	T2	-	T2
43	T3	T4a	T3	T3	T2	T2	T3
44	T3	T3	-	T3	T3	-	T3
45	T4a	T4a	-	T3	T3	-	T3
46	T3	T4a	T3	T3	T3	-	n/a
47	T4a	T4a	-	n/a	n/a	n/a	T4a
48	T3	T3	-	T3	T3	-	T2
49	T3	T3	-	T3	T3	-	T4b
50	T4a	T3	T4a	T3	T3	-	T3
51	T3	T3	-	T3	T3	-	T4a
52	T3	T3	-	T3	T3	-	T3
53	T3	T4a	T3	n/a	n/a	n/a	T4a
54	T3	T3	-	T3	T3	-	T2
55	T3	T3	-	T3	T3	-	T3
56	T3	T3	-	T2	T2	-	n/a
57	T2	T2	-	n/a	n/a	n/a	T1
58	T3	T3	-	T3	T3	-	n/a
59	T3	T3	-	T3	T3	-	T3

60	T3	T3	-	n/a	n/a	n/a	T3

N STAGE										
**Patients**	**MRI**			**CT**			**PET/CT**			**EUS**
	**Reader 1**	**Reader 2**	**Consensus**	**Reader 3**	**Reader 4**	**Consensus**	**Reader 5**	**Reader 6**	**Consensus**	
1	N2	N1	N2	N2	N2	-	N0	N0	-	N2
2	N1	N1	-	N1	N1	-	N1	N1	-	N1
3	N2	N2	-	N2	N2	-	N2	N2	-	N2
4	N0	N2	N2	N2	N2	-	N1	N0	N1	N2
5	N2	N1	N2	N2	N2	-	N1	N2	N2	N2
6	N1	N0	N1	n/a	n/a	n/a	N0	N1	N0	N2
7	N2	N2	-	N1	N1	-	N1	N1	-	N0
8	N2	N2	-	n/a	n/a	n/a	N0	N0	-	N2
9	N0	N0	-	N0	N0	-	N0	N0	-	N0
10	N2	N2	-	N1	N1	-	N1	N1	-	N2
11	N1	N1	-	N1	N1	-	N1	N1	-	N0
12	N1	N1	-	n/a	n/a	n/a	N0	N0	-	N0
13	N0	N0	-	n/a	n/a	n/a	N1	N0	N0	N0
14	N0	N0	-	N0	N0	-	N0	N2	N0	N0
15	N1	N0	N1	N0	N1	N0	n/a	n/a	n/a	N1
16	N2	N1	N2	N1	N1	-	N0	N0	-	N2
17	N2	N2	-	N1	N1	-	N2	N2	-	N2
18	N3	N2	N3	N3	N3	-	n/a	n/a	n/a	N1
19	N0	N1	N1	n/a	n/a	n/a	N0	N0	-	N2
20	N0	N0	-	n/a	n/a	n/a	N0	N0	-	N2
21	N1	N1	-	N1	N1	-	N1	N1	-	N2
22	N2	N2	-	N2	N2	-	N2	N2	-	N3
23	N0	N0	-	N1	N1	-	N1	N0	N0	N2
24	N2	N2	-	n/a	n/a	n/a	N2	N2	-	N2
25	N1	N1	-	N1	N1	-	N0	N0	-	N2
26	N2	N2	-	n/a	n/a	n/a	N3	N3	-	N2
27	N1	N1	-	N2	N2	-	N2	N1	N1	N1
28	N2	N2	-	n/a	n/a	n/a	N1	N1	-	N1
29	N2	N2	-	N2	N2	-	N2	N2	M0	N3
30	N0	N0	-	N0	N0	-	N0	N0	-	n/a
31	N1	N1	-	N1	N1	-	N0	N0	-	N0
32	N2	N2	-	N2	N2	-	N2	N2	-	N2
33	N0	N0	-	N0	N1	N0	N0	N0	-	n/a
34	N2	N3	N3	N3	N3	-	N3	N3	-	N2
35	N1	N0	N1	N1	N1	-	N1	N0	N1	N1

36	N2	N2	-	N2	N2	-	n/a	n/a	n/a	N2
37	N2	N1	N2	N2	N2	-	N1	N2	N2	N1
38	N0	N1	N1	N0	N0	-	N0	N0	-	N2
39	N0	N1	N0	n/a	n/a	n/a	N0	N0	-	N1
40	N2	N2	-	n/a	n/a	n/a	N1	N1	-	N2
41	N3	N3	-	N3	N3	-	N3	N3	-	N2
42	N0	N0	-	N0	N0	-	N0	N0	-	n/a
43	N1	N1	-	N1	N1	-	N0	N0	-	N2
44	N2	N2	-	N2	N2	-	N2	N2	-	N3
45	N1	N1	-	N1	N1	-	N0	N1	N0	N0
46	N1	N0	N1	N2	N1	-	N1	N1	-	n/a
47	N3	N2	N3	n/a	n/a	n/a	N3	N2	N3	N1
48	N1	N1	-	N0	N0	-	N0	N0	-	N0
49	N2	N2	-	N2	N2	-	N0	N2	N1	N+
50	N0	N0	-	N1	N1	-	N0	N0	-	N1
51	N1	N1	-	N1	N1	-	N0	N0	-	N2
52	N1	N1	-	N1	N1	-	N1	N1	-	N1
53	N2	N2	-	n/a	n/a	n/a	n/a	n/a	n/a	N+
54	N1	N1	-	N1	N1	-	N0	N1	N1	N1
55	N2	N2	-	N1	N2	N1	N0	N2	N2	N1
56	N0	N0	-	N1	N1	-	N0	N0	-	n/a
57	N0	N0	-	n/a	n/a	n/a	N1	N0	N1	N0
58	N2	N2	-	N2	N2	-	n/a	n/a	n/a	n/a
59	N2	N2	-	N1	N1	-	N1	N1	-	N1
60	N1	N2	N1	n/a	n/a	n/a	N1	N1	-	N2

M STAGE									
Patients	MRI			CT			PET/CT		
	Reader 1	Reader 2	Consensus	Reader 3	Reader 4	Consensus	Reader 5	Reader 6	Consensus
1	M0	M0	-	M0	M0	-	M0	M0	-
2	M0	M0	-	M0	M0	-	M0	M0	-
3	M0	M0	-	M0	M0	-	M0	M0	-
4	M1	M1	-	M0	M0	-	M1	M1	-
5	M0	M0	-	M0	M0	-	M0	M0	-
6	M0	M0	-	n/a	n/a	n/a	M0	M0	-
7	M0	M0	-	M0	M0	-	M0	M0	-
8	M0	M0	-	n/a	n/a	n/a	M0	M0	-
9	M0	M0	-	M0	M0	-	M0	M0	-
10	M1	M1	-	M1	M1	-	M1	M1	-
11	M0	M0	-	M0	M0	-	M0	M0	-

12	M0	M0	-	n/a	n/a	n/a	M0	M0	-
13	M0	M0	-	n/a	n/a	n/a	M0	M0	-
14	M0	M0	-	M0	M0	-	M0	M0	-
15	M0	M0	-	M0	M0	-	n/a	n/a	n/a
16	M0	M0	-	M0	M0	-	M0	M0	-
17	M0	M0	-	M0	M0	-	M0	M0	-
18	M0	M0	-	M0	M0	-	n/a	n/a	n/a
19	M0	M0	-	n/a	n/a	n/a	M0	M0	-
20	M0	M0	-	n/a	n/a	n/a	M0	M0	-
21	M0	M0	-	M0	M0	-	M0	M0	-
22	M0	M0	-	M0	M0	-	M0	M0	-
23	M0	M0	-	M0	M0	-	M0	M0	-
24	M0	M0	-	n/a	n/a	n/a	M0	M0	-
25	M0	M1	M0	M0	M0	-	M0	M0	-
26	M0	M0	-	n/a	n/a	n/a	M0	M0	-
27	M0	M0	-	M0	M0	-	M0	M1	M0
28	M0	M0	-	n/a	n/a	n/a	M0	M0	-
29	M1	M1	-	M0	M0	-	M0	M1	M0
30	M0	M0	-	M0	M0	-	M0	M0	-
31	M0	M0	-	M0	M0	-	M0	M0	-
32	M0	M0	-	M0	M0	-	M0	M0	-
33	M0	M0	-	M0	M0	-	M0	M0	-
34	M0	M0	-	M0	M0	-	M0	M0	-
35	M0	M0	-	M0	M0	-	M0	M0	-
36	M0	M0	-	M0	M0	-	n/a	n/a	n/a
37	M0	M0	-	M0	M0	-	M0	M0	-
38	M0	M0	-	M0	M0	-	M0	M0	-
39	M0	M0	-	n/a	n/a	n/a	M0	M0	-
40	M0	M0	-	n/a	n/a	n/a	M0	M0	-
41	M1	M1	-	M1	M1	-	M1	M1	-
42	M0	M0	-	M0	M0	-	M0	M0	-
43	M0	M0	-	M0	M0	-	M0	M0	-
44	M0	M0	-	M0	M0	-	M0	M0	-
45	M0	M0	-	M0	M0	-	M0	M0	-
46	M0	M0	-	M0	M0	-	M0	M0	-
47	M1	M1	-	n/a	n/a	n/a	M1	M1	-
48	M0	M0	-	M0	M0	-	M0	M0	-
49	M0	M0	-	M0	M0	-	M0	M0	-
50	M0	M0	-	M0	M0	-	M0	M0	-
51	M0	M0	-	M0	M0	-	M0	M0	-
52	M0	M0	-	M0	M0	-	M0	M0	-

53	M0	M0	-	n/a	n/a	n/a	n/a	n/a	n/a
54	M0	M0	-	M0	M0	-	M0	M0	-
55	M0	M0	-	M0	M0	-	M0	M0	-
56	M0	M0	-	M0	M0	-	M0	M0	-
57	M0	M0	-	n/a	n/a	n/a	M0	M0	-
58	M0	M1	M1	M0	M0	-	n/a	n/a	n/a
59	M0	M0	-	M0	M0	-	M0	M0	-
60	M0	M0	-	n/a	n/a	n/a	M0	M0	-
